# Study protocol for identification of patients with risk of cognitive impairment in advanced pharmaceutical care in a community pharmacy

**DOI:** 10.3389/fpubh.2025.1606381

**Published:** 2025-08-12

**Authors:** Zuzana Macekova, Michaela Krivosova, Nikola Hudakova, Jozef Dragasek, Michal Hajduk, Viera Zufkova, Jan Klimas, Miroslava Snopkova

**Affiliations:** ^1^Department of Pharmacy and Social Pharmacy, University of Veterinary Medicine and Pharmacy in Kosice, Kosice, Slovakia; ^2^Biomedical Centre Martin, Jessenius Faculty of Medicine in Martin, Comenius University in Bratislava, Martin, Slovakia; ^3^Department of Morphological Disciplines, University of Veterinary Medicine and Pharmacy in Kosice, Kosice, Slovakia; ^4^1st Department of Psychiatry, Faculty of Medicine, Pavol Jozef Safarik University and University Hospital, Kosice, Slovakia; ^5^Department of Psychology, Faculty of Arts, Comenius University in Bratislava, Bratislava, Slovakia; ^6^Department of Languages, Faculty of Pharmacy, Comenius University in Bratislava, Bratislava, Slovakia; ^7^Department of Pharmacology and Toxicology, Faculty of Pharmacy, Comenius University in Bratislava, Bratislava, Slovakia; ^8^Department of Organisation and Management of Pharmacy, Faculty of Pharmacy, Comenius University in Bratislava, Bratislava, Slovakia

**Keywords:** clinical pharmacy services, cognitive impairment, pharmacist-led screening, community pharmacy, dementia prevention in primary care, physician-pharmacist cooperation

## Abstract

**Introduction:**

Cognitive impairment (CI) is a growing public health problem. Our study is based on the fact that cognitive assessment in community pharmacy focused on early identification of undiagnosed CI has received limited attention. As pharmacists are the most accessible health professionals due to the availability of community pharmacies to the public, they have the potential to bring improvement in this area. Early identification of at-risk patients with CI by performing cognitive testing within advanced pharmaceutical care may improve the availability of targeted physician-indicated treatment.

**Methods and analysis:**

The study is a multicentric study that will include cognitive screening within pharmaceutical care. We will use the Slovak version of the short form of the Montreal Cognitive Assessment (s-MoCA) test. Study participants will be at-risk patients undergoing cognitive screening in community pharmacies. Secondarily, we will evaluate the risk factors related to CI, such as at-risk medication use and modifiable dementia risk factors (e.g., cardiovascular and mental comorbidities, aging, and lifestyle habits).

**Ethics and dissemination:**

This study was approved by the Ethics Committee of the Faculty of Pharmacy, Comenius University Bratislava (Ethics Committee Statement 01/2024). All procedures follow the relevant guidelines and regulations and the Declaration of Helsinki.

## 1 Introduction

### 1.1 Epidemiology and cognitive screening

Cognitive disorders represent a growing public health concern given the increasing number of seniors reaching an at-risk age (1, 2), associated with a subjectively or objectively measurable decline in at least one of the domains of cognitive functions, such as verbal abilities, spatial orientation, episodic memory, processing speed and executive functions (3). Only limited attention has been given to cognitive assessment in the community pharmacy targeting the early identification of undiagnosed cognitive impairment (CI) (4–9). As pharmacists are the most accessible health professionals due to the availability of community pharmacies to the public, they have the potential to be involved in early identification of at-risk patients who have not yet received a professional examination by a physician (4). Dementia and Alzheimer’s disease are the most common cognitive disorders affecting older people aged 60+ (1, 10), with a globally devastating impact on society with a disproportionate burden on health, economic and social care (11–13). Therefore, prevention, diagnosis, and pharmacological and non-pharmacological treatment of dementia are among the international priorities of the World Health Organization (WHO) (1, 2). It is essential to recognize CI at a reversible stage, and early intervention and appropriate interventions can prevent the development of dementia. This stage called mild cognitive impairment (MCI), is a transitional phase between cognitive ability decline due to age and the terminal stage of CI, dementia. Mental health and memory problems are crucial issues with significant impact on life quality. They can also affect patients’ level of medication adherence and self-care ability, which can result in poor long-term health outcomes (5, 8). Secondarily, cognitive decline may also be accelerated by the use of at-risk medications, especially when taking them for a long time (14, 15). These drugs suppress the cholinergic neurotransmitter pathways affecting brain structures such as the hippocampus and the neocortex, which are already vulnerable to age-related CI (16). Analysis of chronic pharmacotherapy can help identify at-risk medications and suggest a safe alternative, if possible. Anticholinergics, sedatives and benzodiazepines are the commonly used drugs with adverse effects on cognitive function (15–18). When they are combined, their adverse effect is cumulative (15, 19). A well-used tool is an assessment of the anticholinergic burden (ACB) scale (17). Implementing cognitive screening for early identification of patients with CI into pharmaceutical care can forward these patients to a doctor without undue delay and allow early initiation of their treatment and monitoring of further cognitive deterioration. Moreover, treatment of CI could reduce the risk of non-adherence to pharmacotherapy for already diagnosed chronic diseases (5, 20). Community pharmacists are suitable for preventing dementia by implementing cognitive screening within advanced pharmaceutical care (5, 20). Early identification of at-risk patients with CI could improve the availability of physician-indicated targeted treatment.

Establishing a diagnosis for CI requires a comprehensive approach and an overall assessment of the patient’s cognitive performance using multiple diagnostic tools and is strictly the physician’s responsibility (21). Brief screening tests such as the Mini-Mental State Examination (MMSE) (22), the Clock Drawing Test (CDT) (23), and the Montreal Cognitive Assessment (MoCA) are used for an indicative examination of cognitive abilities. The MoCA test is a gold standard, one of the most commonly used cognitive screening tools worldwide. It is characterized by a high sensitivity and specificity for MCI (21), and short and standard versions of the MoCA appear to be effective in identifying CI (24). It was translated into many languages, including Slovak (25, 26). Its time-saving short version for pharmacy practice is suitable (27–29). If poorer cognitive abilities are noted in an orientation cognitive screening within pharmaceutical care, it does not mean the patient suffers from CI. A suspected CI is suggested. A physician’s assessment of the patient’s cognitive status is needed to confirm the CI (21).

### 1.2 CI risk factors

**Figure 1:**
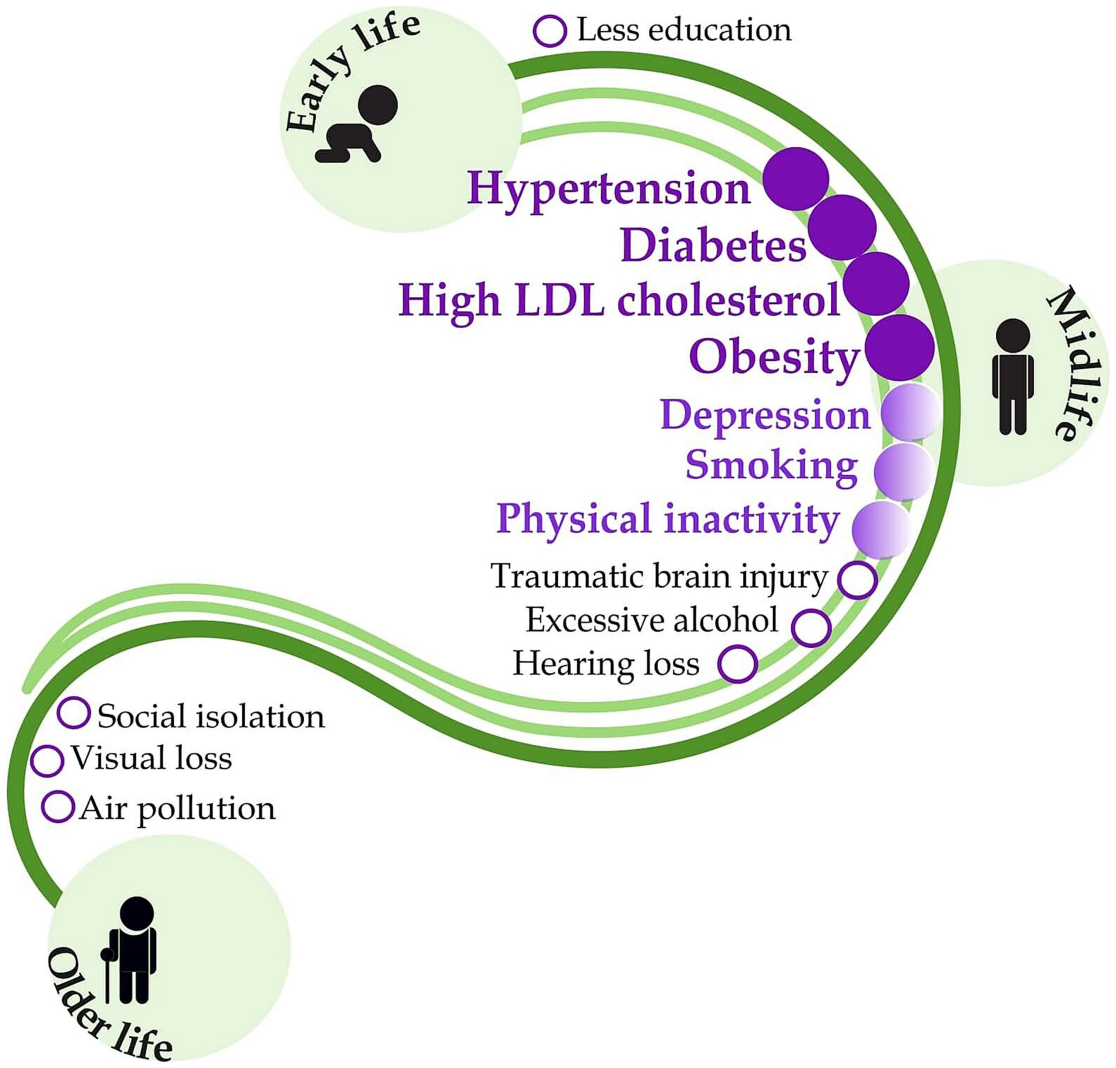
Modifiable risk factors for dementia and the potential role of pharmacists in their management. Dark purple indicates routinely performed pharmaceutical interventions with a well-documented patient outcome. Light purple shows interventions with poor outcome evidence, and the black letters indicate risk factors outside the pharmacist’s competence. Figure was adapted from the original figure by Livingston et al. (12) with permission from Elsevier LTD., which has been expanded to include information regarding the potential role of pharmacists in their management according to Macekova et al. (34), using Inkscape graphics editor.

The development of CI and dementia is related to the interaction of many factors. Nowadays, potentially modifiable factors are well-known (12) (Figure 1). Cardiovascular risk factors are together with Aging and the Incidence of Dementia (CAIDE) expressed by the CAIDE Dementia Risk Score (30). They can evaluate a patient’s risk of developing dementia (31). Most of these factors of CI, such as hypertension, obesity, diabetes and high cholesterol, can be effectively influenced by pharmacists’ interventions. At present, pharmacists’ competencies are expanding and include the management of chronic diseases and medication safety (32), assessment of potentially inappropriate medication use in the older adult (33), monitoring biochemical parameters, blood pressure measurement, obesity management, smoking cessation and others, all of which contribute to the individualisation of pharmaceutical care that is more patient-centered (34). Chronic cardiovascular disease and metabolic syndrome (MetS) are among the modifiable risk factors for dementia. Therefore, attention should be paid to monitoring cognitive abilities in patients suffering from these conditions. CI may lead to non-adherence to treatment, which in turn may result in the development of dementia (20, 35). Advanced pharmaceutical care now routinely includes the management of most modifiable risk factors for CI and dementia. This is a relevant basis for the subsequent expansion of pharmaceutical care targeting early recognition of CI through simple cognitive screening as a pharmaceutical service.

### 1.3 Role of pharmacists

Pharmacists can support the management of most patients suffering from diseases that do not require a visit to the doctor, thus reducing the burden of medical care. Consequently, physicians can focus on patients with severe disorders (36). In addition, pharmacists can play a role in managing patients suffering from chronic diseases (37), including cognitive disorders (6, 8, 9, 38). Nevertheless, it is still uncertain whether these pharmaceutical interventions bring adequate benefits to the patients and improve their health status. The patient’s cognitive state may be the limitation for achieving satisfactory results, as in the case of CI, the patient needs an individual, specific approach in the provision of health care, including pharmaceutical care. For this reason, our study aimed to develop a standard protocol for assessing cognitive screening of patients aged 50 years and over in community pharmacies so that the pharmacist can adapt this approach to the patient in the provision of pharmacy care.

## 2 Methods and analysis

### 2.1 Design and setting

**Figure 2:**
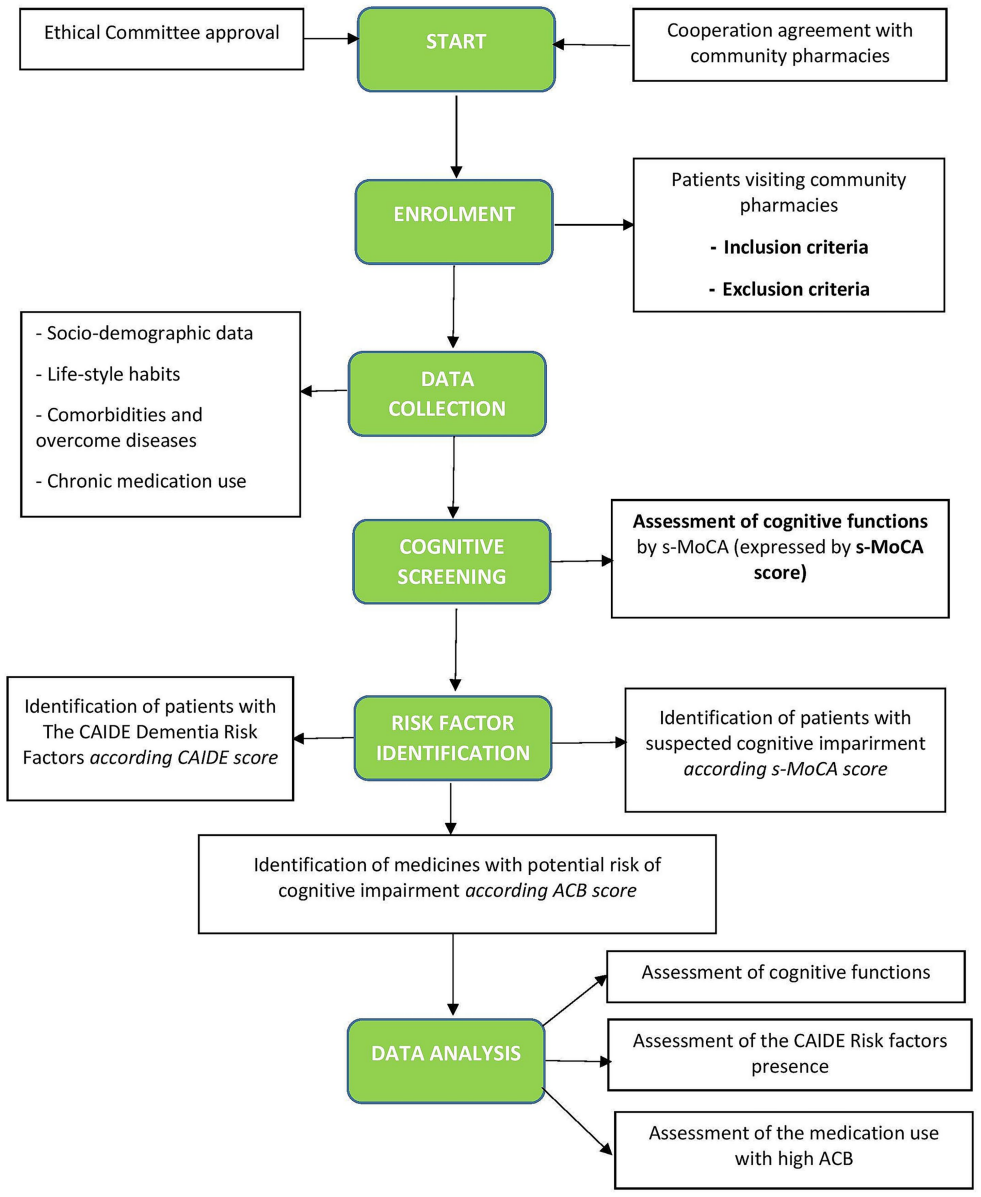
Flowchart of the study. CAIDE, Cardiovascular Risk Factors, Aging, and Incidence of Dementia (The CAIDE Dementia Risk Score), s-MoCA, short version of the Montreal Cognitive Assessment Screening Tool; ACB, Anticholinergic Burden Scale.

We designed a study protocol for a prospective observational clinical cohort study in Slovakia to evaluate multifactorial and specific risk factors for CI in the adult population aged 50+ as a part of advanced pharmaceutical care in community pharmacies (Figure 2). The current study protocol is developed based on our previous findings (39, 40) and offers an advanced method of identifying patients at risk for CI by pharmacist intervention. Firstly, we focus on performing a cognitive screening using a short version of the Montreal Cognitive Assessment (21, 28). Secondly, we will assess the risk of developing dementia according to the CAIDE Dementia Risk Score, which includes lifestyle factors, age, and comorbidities altogether expressed as the CAIDE score (31). Third, we will assess the risk of medication use by impacting cognitive health according to the ACB scale using the free available online ACB calculator (41).

### 2.2 Cohort and sample size calculation

Adult patients aged 50 years or older receiving pharmaceutical care (patients who visit community pharmacy for different reasons, e.g., to collect a medication with/without prescription, pharmaceutical counseling, etc.) in community pharmacies in Slovakia who can complete the questionnaires and are willing to participate in the study will be included. Inclusion and exclusion criteria are shown in Table 1. Exclusion criteria also include disorders which can be related to cognitive dysfunction (e.g., schizophrenia). Pharmacy clients will receive written information about the study objectives, and consent will be obtained to confirm participation. By their signature, they will also agree to participate only once (to avoid duplicities). Individuals will be informed that participation is voluntary and can be terminated at any time. Also, they will be ensured that access to the collected data will be restricted to the study investigators only. The minimum sample size for our analysis was based on the estimated proportion. The sample size was set using an online sample size calculator for a 95% confidence interval and a 5% margin of error (42), according to previously used in Kosirova et al. (33). The minimum number of included subjects was estimated to be 385 according to the total number of adults aged 50 and over in 2023 (*N* = 2,033,555); (43).

**Table 1:** Inclusion and exclusion criteria.

Inclusion criteria	Exclusion criteria
Adults aged 50 and older, patients with type 2 diabetes mellitus without age limitation Undergoing at least one counseling by a pharmacist No previous diagnosis of CI/dementia as determined by ICD-10 codes or the presence of a prescription for anti-dementia medications (cholinesterase inhibitors or memantine) Ability to consent to participate in the study Ability to communicate in Slovak	Adults outside the age criteria Adults in permanent senior care or nursing home A serious mental illness such as schizophrenia code ICD-10 and targeting medication A pre-existing diagnosis of CI/dementia

### 2.3 Data collection

Data will be collected by trained pharmacists working in community pharmacies. They will be recorded on a pre-printed study form (socio-demographic data, lifestyle habits, evidence of comorbidities, and family history of Alzheimer’s disease and dementia). Table 2 summarizes all items that will be recorded.

**Table 2:** Basic characteristics and risk factors of the study population.

Socio-demographic features and lifestyle habits	
Age	Number of years
Gender	Multiple choice (*Female/Male*)
Education level	Number of school years
Waist circumference	Number (*cm*)
Physical activity habits	Open-ended questions (*frequency, duration per week, type of physical activity*)
Marital status	Multiple choice (*single/married/widow/divorced*)
Live in the household	Multiple choice (*alone/with other family members*)
Sleep duration	Open-ended questions (*frequency and duration per night/day*)
Smoking habits	Number of cigarettes per day
Alcohol consumption	Number and type of drinks per day
Comorbidities	
Hypertension	Yes/No
Dyslipidaemia	Yes/No
Type 2 diabetes	Yes/No
Overcome diseases	
Stroke	Yes/No
Thrombosis	Yes/No
Head injury	Yes/No
Actual and/or past problems	
Memory problems	Yes/No
Vision problems	Yes/No
Hearing problems	Yes/No
Smell problems	Yes/No
Family history of Alzheimer’s disease and/or dementia pathology (in line of parents, grandparents)	Yes/No

### 2.4 Place and staffing requirements

The screening site will be community pharmacies with suitable cognitive screening space. A suitable space may be a separate room in the pharmacy or a separate part of another area to guarantee confidentiality for the patient. Only trained pharmacists or pharmacy students under the supervision of a trained pharmacist may perform the screening. The pharmacist enrolled in the study must also provide the name and address of the pharmacy where the screening will be conducted.

Participating pharmacists will be educated through a 2-h webinar followed by a workshop with a pharmacists´ competency assessment, according to Rickles and colleagues (8), which will be prepared and implemented with the cooperation of the Slovak Chamber of Pharmacists, the Faculty of Pharmacy of Comenius University Bratislava, the University of Veterinary Medicine and Pharmacy in Kosice and medical societies. Trained pharmacists will be trained in using cognitive screening tools and the MoCA Test, receive information on modifiable risk factors for dementia and early warning signs, and gain practical experience in strategies to identify at-risk patients within pharmaceutical care. The training program will also provide a detailed overview of study consent forms and documents, an overview of the patient care process, dementia risk assessment, and an overview of protocols for patient follow-up, data collection, and reporting.

### 2.5 Personal competence

1. Collecting data and performing cognitive screening – trained pharmacists or pharmacy students under the supervision of a trained pharmacist.
2. Interpretation of results – trained pharmacists only.
3. Patient outcomes should be communicated to the physician–pharmacists only.

#### 2.5.1 The target group of individuals for which the screening is recommended

1. Aged 50+.
2. Suffering from cardiovascular disease and/or 2 type diabetes mellitus (in case of DM2 without age limitation).
3. With dementia risk factors according to the CAIDE score.
4. With a family history of cognitive disorders (MCI, dementia, Alzheimer’s disease).
5. With at least one of the Alzheimer’s disease international early warning signs.

### 2.6 Cognitive screening

The gold standard screening tool, the Montreal Cognitive Assessment (MoCA), one of the available cognitive screening instruments, scans seven cognitive domains: executive functioning; visuospatial abilities; language; attention, concentration and working memory; abstract reasoning; memory and orientation, will be used. We use the short variant (s-MoCA) (28, 29) in the Slovak language, presenting a comparable alternative for detecting MCI and dementia. This short variant consists of 8 items. These items measure visuospatial and executive functions (trail making and clock drawing), language abilities (animal naming – rhinoceros), attention (serial 7 s – counting by subtracting seven), verbal fluency (naming for 1 min), abstraction (watch), delayed recall of words, orientation (place) (29). According to our previous outcomes, this simple, shortened version is an easy-to-use cognitive screening. It is an applicable compound of pharmaceutical care for adult patients aged 50 and older with cardiovascular disease and/or suspected metabolic syndrome (sMetS) to explore their cognitive state. This shortened form of the MoCA scale has a range of 0–16 points; the time of completion is 5–7 min (which is only one-third the length of the original MoCA), and a cut-off of ≤12 represents a cognitive impairment (29).

### 2.7 Assessment of dementia risk factors

The CAIDE scoring system will be applied to the occurrence of modifiable risk factors (31). It was designed to determine the risk of developing dementia in cognitively intact individuals aged 40 to 65 years. This tool assesses common, easy-to-obtain, measurable data such as age, sex, education, blood pressure, cholesterol level, BMI, and physical activity. Each item is scored, and the resulting score represents the level of risk of developing cognitive impairment or dementia. The cut-off score represents 8–9 points for low and normal risk. Higher scores (10–15) indicate an increased risk of developing cognitive impairment (31).

We will also assess an occurrence of the suspected MetS (sMetS) as it can be easily identified in community pharmacy and may affect cognitive function (40, 44). The presence of sMetS will be estimated according to the International Diabetes Federation Worldwide Definition of MetS (45), 2005, modified for the European population (46). Accordingly, patients will be divided into groups according to the presence or absence of sMetS (sMetS+; sMetS-), and we will compare cognitive abilities in the test s-MoCA between two subpopulations (sMetS+/sMetS-) (40).

### 2.8 Analysis of chronic medication

Medication analysis will be focused on the identification of at-risk medication use with an anticholinergic burden risk used in included participants. We will assess the cumulative effect of medication with anticholinergic properties taking long-term, which can adversely impact cognitive performance and physical abilities and increase the mortality risk (15). We will use an expert opinion-derived risk scale, the ACB Scale, which helps quantify the risk of anticholinergic burden (17). The list of at-risk medications is summarized in Supplementary Table 1. The scale ranks the anticholinergic activity of drugs into four categories, according to their anticholinergic effect and potential for impairment of cognition: (i) no anticholinergic activity, ACB = 0; (ii) possible anticholinergic activity, ACB = 1; (iii) definite anticholinergic activity, ACB = 2; and (iv) definite high anticholinergic activity, ACB = 3 (15). Medication with the scores 1, 2 and 3 can be found in Supplementary Table 1. If the medication is not displayed in the list, its ACB score equals 0. In patients taking more than one medication from the list, scores are cumulative, and a total score ≥3 means a high risk for cognitive impairment. Each one-point increase in the ACB total score is associated with an apparent decline in the MMSE score (16).

### 2.9 Patient counseling

**Figure 3:**
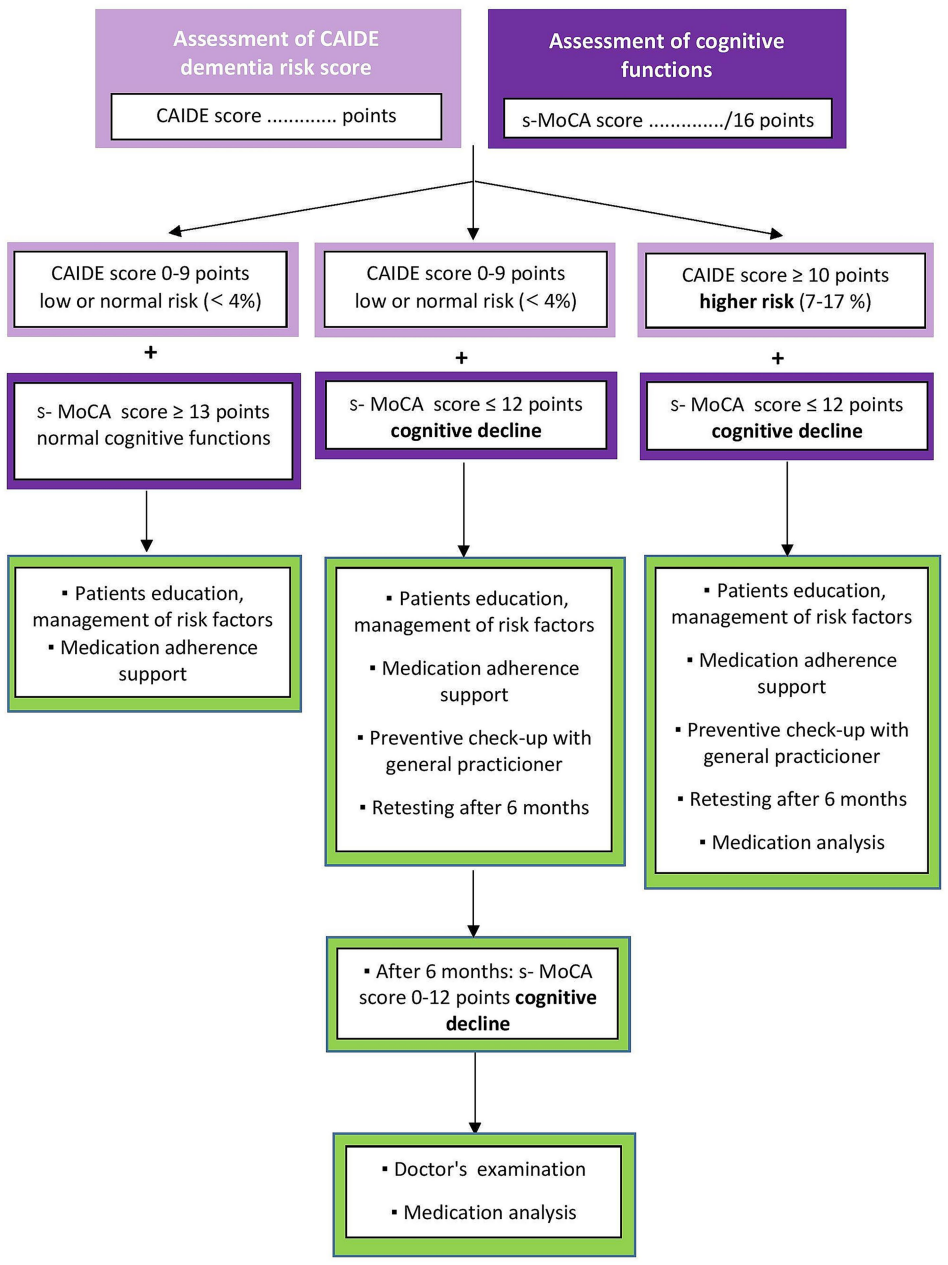
Analysis plan – counseling between pharmacist and patient.

Pharmaceutical counseling will be provided to patients concerning their needs, family and personal history and comorbidities or even physician’s recommendation (Supplementary Table 2). The analysis plan is shown in Figure 3. During the interpretation of the results of cognitive screening (following initial screening or, if necessary, after retesting 6 months later, by the patient’s consent and an agreed appointment with the pharmacist based on a call), it is essential to explain to the patients that this simple cognitive screening is not a substitute for a medical examination and that the result of the screening is not a physician’s diagnosis. Next, the pharmacist will provide a patient’s education about other factors that may influence cognitive decline (such as stress, lifestyle, sleep disturbances, depression and certain medications or substance use, e.g., caffeine, alcohol). Patients will get printed educational materials about risk factors for cognitive impairment and early warning signs of dementia.

According to results of screening and pharmacist’s consideration, patients may be referred to a physician if their condition requires it. In such cases, the pharmacist will use the uniform information form for the physician (Supplementary Table 3). The patient receives an examination report informing the physician of the findings.

### 2.10 Physician referral

**Figure 4:**
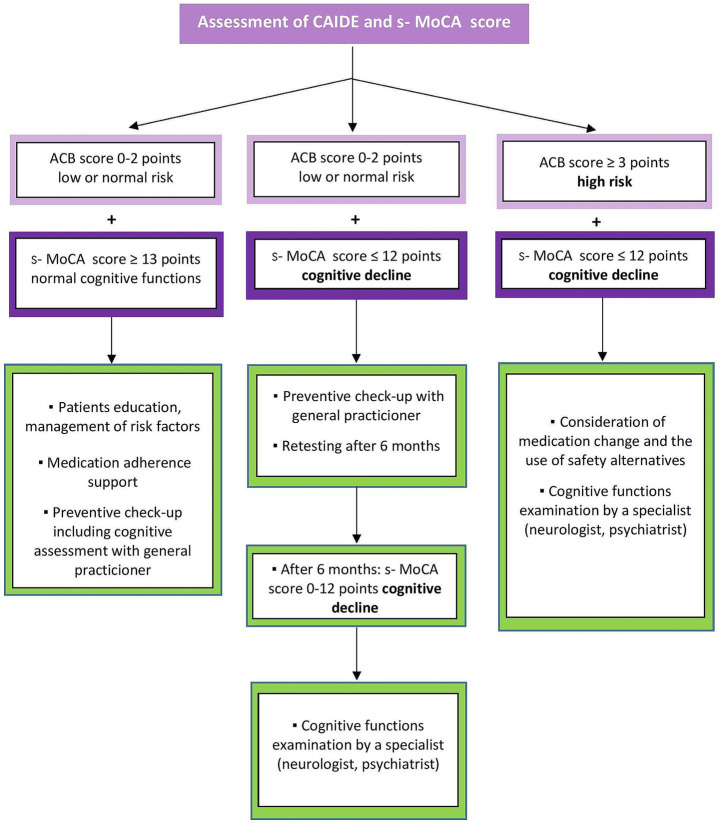
Analysis plan – counseling between pharmacist and physician.

For each patient, the pharmacist will specifically consider whether their condition requires a visit to the doctor or a pharmacy consultation is sufficient. The analysis plan is shown in Figure 4. Specific groups of patients are referred to a physician if they score decreased cognitive abilities on the s-MoCA test. Patients:

- with memory problems,
- with at least one of the early signals of dementia,
- at risk of developing dementia, according to CAIDE,
- that do not have regular preventive check-ups,
- that are long-term users of at-risk medication (Supplementary Table 1),
- with long-term use of over-the-counter memory aids.

The result of the pharmacotherapy analysis will be received only by a doctor. The patient will not be given the results to maintain adherence. Every patient in the study should be referred to a general practitioner for a preventive check-up if they have not had one in the last 2 years. During follow-up, the pharmacist will contact patients electronically to obtain feedback on whether they have addressed their condition with a doctor and whether the presence of CI has been confirmed.

## 3 Outcomes

In this study, we focus on a cohort of adults aged 50+ with lower cognitive abilities expressed by s-MoCA scores (≤ 12 points). These patients will be defined as those with suspected CI (CI+) who will be referred for medical examination. We will then record whether a physician has confirmed our suspicion of CI.

In the CI+ group, we will collect data on the prevalence of CI risk factors (expressed by CAIDE score) and the use of risk medications (expressed by ACB score) and then compare them with a control group.

## 4 Statistical analyses and mitigation of bias

The obtained data will be analyzed using GraphPad, version 8.0.1 (GraphPad Prism, San Diego, CA, USA) and the SAS Education Analytical Suite for Microsoft Windows, version 9.3 (SAS Institute Inc., Cary, NC, USA). We will perform fundamental descriptive analyses (calculation of mean and SD), normality tests for the included variables, correlation tests, *t*-tests for two independent variables, and one-way analysis of variance (ANOVA) in the case of more independent groups. Multivariate regression models will be used to compare cognitive outcomes across subgroups.

Since there is no randomization or intervention control in this study, several types of bias can affect validity. In the study, we will mitigate selection bias through design-stage (broad and inclusive recruitment, clear criteria, consistent enrolment) and analysis-stage strategies (statistical adjustment, sensitivity analyses). Observer bias will be mitigated through standardization, pharmacist training, objective measurements, and data monitoring strategies. Social desirability bias will be mitigated through use of questionnaire design (e.g., using neutral wording and validated scales) and through pharmacist training with a focus on his/her empathy and neutrality.

## 5 Discussion

### 5.1 Objectives and hypotheses

#### 5.1.1 Study objectives

1. To perform a standardized cognitive screening by a trained pharmacist within advanced pharmaceutical care in a community pharmacy.
2. To test the association between the presence of modifiable Cardiovascular Risk Factors, Aging, and Incidence of Dementia (according to The CAIDE Dementia Risk Score) and poorer cognitive abilities (expressed s-MoCA score) in the adult population aged 50+.
3. To assess the medication use related to CI by medication analysis as a part of pharmaceutical counseling in a community pharmacy.

#### 5.1.2 Hypotheses

According to our previous results, we hypothesize:

1. Short cognitive screening, a part of advanced pharmaceutical care, can help identify patients who need further cognitive evaluation by a general practitioner or a specialist.
2. Analysis of pharmacotherapy can help to identify at-risk medication use related to CI.

Pharmacists in a community pharmacy could help monitor modifiable risk factors of CI.

### 5.2 Strengths and limitations of this study

- The study focuses on at-risk patients with CIs within pharmaceutical care in a community pharmacy. Implementing cognitive screening in the pharmacy setting may contribute to the early identification of patients with CI, reducing the pressure on ambulatory care associated with CI prevention.
- Data will be collected using easy-to-use cognitive screening by a pharmacist.
- The realization of cognitive screening, correct evaluation of results and interpretation, and the pharmacist’s final decision about referring a patient to a physician will depend on their experiences and critical judgment. Hence, the education of pharmacists in their training program is a crucial stage of this study. It is also important to consider the patient’s other comorbidities, for instance, whether there exists a bidirectional relationship between depressive disorder and cognitive deficits, which may affect the outcome of screening tests. The exclusion of institutionalized older adults may limit the generability of the study cohort.
- Pharmacists must be able to communicate outcomes concerning patients’ conditions. Collaboration between pharmacists and physicians is important because a patient with suspected CI identified by a pharmacist cannot be diagnosed with CI without the doctor’s confirmation.
- Furthermore, various tools can characterize the anticholinergic burden score differently. While one tool can classify drugs such as clopidogrel, furosemide, and zolpidem as potentially anticholinergic, according to another tool (e.g., the ACB Calculator), they have no increased risk.
