# Supplementary material for: Study protocol for identification of patients with risk of cognitive impairment in advanced pharmaceutical care in a community pharmacy
Source: Front Public Health. 2025 Aug 12;13:1606381. doi: 10.3389/fpubh.2025.1606381 (PMC12379007; doi:10.3389/fpubh.2025.1606381)
Supplement: Supplementary file 1 [file Table_1.docx]

**Supplementary Table 1:** Medicines according to Anticholinergic burden scale (ACB score system), https://www.acbcalc.com [ACB Calculator](https://www.acbcalc.com/)

| **ACB 1** | | | | | |
| --- | --- | --- | --- | --- | --- |
| Alimemazine | Chloroquine | Entacapone | Isosorbide | Nitrazepam | Rotigotine |
| Alprazolam | Chlortalidone | Ergotamine | Isosorbide dinitrate | Nizatidine | Selegiline |
| Alverine | Ciclosporin | Escitalopram | Isosorbide mononitrate | Olopatadine ophtalmic | Sertraline |
| Amantadine | Cimetidine | Etoricoxib | Ketotifen ophthalmic | Omeprazole | Sumatriptan |
| Aminophylline | Citalopram | Fentanyl | Lansoprazole | Oxazepam | Temazepam |
| Ampicillin | Clindamycin | Fentanyl topical | Levocetirizine | Oxycodone | Theophylline |
| Aripiprazole | Clonazepam | Flunitrazepam | Levodopa | Pancuronium | Tiagabine |
| Azenapine | Clorazepate | Fluoxetine | Lithium | Pantoprazole | Tiotropium |
| Azelastine nasal | Corticosterone | Fluphenazine | Loperamide | Pheniramine ophthalmic | Tobramycin |
| Azelastine ophthalmic | Cortisone | Flurazepam | Loratadine | Phenobarbitol | Trandolapril |
| Barberry | Cycloserine | Fluvoxamine | Lorazepam | Piperacillin | Tranylcypromine |
| Bromocriptine | Cyclosporine | Gentamicin | Meclizine | Pramipexole | Triamcinolone |
| Bupropion | Desloratadine | Glycopyrronium – inhaled | Metformin | Prednisolone | Triamterene |
| Captopril | Desvenlafaxine | Guaifenesin | Methadone | Procainamide | Triazolam |
| Carbidopa/levodopa | Dexamethasone | Haloperidol | Methylprednisolone | Prochlorperazine | Umeclidinium |
| Cefamandole | Dextromethorphan | Hydralazine | Midazolam | Quinidine | Valproic acid |
| Cefoxitin | Diazepam | Hydrocortisone | Mirtazapine | Ranitidine | Vancomycin |
| Celecoxib | Digitoxin | Hydroxyzine | Morphine | Risperidone | Venlafaxine |
| Cephalothin | Digoxin | Iloperidone | Nalbuphine |  | Ziprasidone |
| Cetirizine | Dimetindene | Ipratropium | Naratriptan |  | Zolmitriptan |
| Chlordiazepoxide | Diphenhydramine crm. | Ipratropium nasal | Neomycin |  | Zuclopenthixol |
| **ACB 2** | | | | | |
| Disopyramide | Maprotiline | Methotrimeprazine | Opipramol | Pethidine | Tramadol |
| Homatropine ophtalmic | Mesoridazine | Nefopam | Perphenazine | Pimozide | Trifluoperazine |
| Loxapine |  |  |  |  |  |
| **ACB 3** | | | | | |
| Amitriptyline | Chlorprothixene | Dicycloverine | Hyoscyamine | Phenindamine | Scopolamine topical |
| Amoxapine | Clemastine | Dimenhydrinate | Imipramine | Pheniramine | Solifenacin |
| Atropine | Clidinium | Diphenhydramine | Ketamine |  |  |
| Azatadine | Clomipramine | Dosulepin | Levomepromazine | Phenyltoloxamine | Thioridazine |
| Belladonna | Clozapine | Doxepin | Methocarbamol | Procyclidine | Tolterodine |
| Benztropine | Cyclizine | Doxylamine | Methscopolamine | Promethazine | Triflupromazine |
| Biperiden | Cyclobenzaprine | Fesoterodine | Nortriptyline | Propantheline | Trihexyphenidyl |
| Brompheniramine | Cyproheptadine | Flavoxate | Olanzapine | Propiverine | Trimethobenzamide |
| Buclizine | Darifenacin | Glycopyrronium injectable | Orphenadrine | Protriptyline | Trimipramine |
| Carbinoxamine | Desimipramine | Homatropine | Oxybutinin | Pyrilamine | Triprolidine |
| Chlorphenamine | Dexbrompheniramine | Hyoscine butylbromide | Paroxetine | Quetiapine | Trospium |
| Chlorpromazine | Dexchlorpheniramine | Hyoscine hydrobromide | Pericyazine | Scopolamine |  |
