# Supplementary material for: Study protocol for identification of patients with risk of cognitive impairment in advanced pharmaceutical care in a community pharmacy
Source: Front Public Health. 2025 Aug 12;13:1606381. doi: 10.3389/fpubh.2025.1606381 (PMC12379007; doi:10.3389/fpubh.2025.1606381)
Supplement: Supplementary file 2 [file Table_2.docx]

**Supplementary Table 2:**

**Physician´s recommendation for cognitive function assessment by pharmacist**

Patient´s name: ............................................................................................................................

Date of birth: ............................................................................................................................

Date of the last preventive check-up in general practitioner: ..................................................................................

Comorbidities (disorders + ICD-10 codes):

........................................................................................................................................................................................................................................................................................................................................................................................................................................................................................................................................................................................................................................................................................................................................................................................................................................................................................................................................................................................................................................................................................................................................................................................................................................................................................................................................................................................................................................................................................................

Medication list:

........................................................................................................................................................................................................................................................................................................................................................................................................................................................................................................................................................................................................................................................................................................................................................................................................................................................................................................................................................................................................................................................................................................................................................................................................................................................................................................................................................................................................................................................................................................

**󠅗 Requirement for assessment of cognitive functions**

**Physician´s requirement date:** ..................................................................

**Physician´s name:**  ..................................................................

**Physician´s signature and stamp:** ..................................................................
