# Supplementary material for: Study protocol for identification of patients with risk of cognitive impairment in advanced pharmaceutical care in a community pharmacy
Source: Front Public Health. 2025 Aug 12;13:1606381. doi: 10.3389/fpubh.2025.1606381 (PMC12379007; doi:10.3389/fpubh.2025.1606381)
Supplement: Supplementary file 3 [file Table_3.docx]

**Supplementary Table 3:**

**Pharmaceutical counselling data between a pharmacist and a physician**

**Patient´s name:**

**Date of birth:**

**Outcomes:**

| **Parameters (points)** | **Value (points)** | **Notes** |
| --- | --- | --- |
| **Total CAIDE score** |  |  |
| **Total ACB score** |  |  |
| **Medicines, according to ACB value** | | |
| ACB=1 |  |  |
| ACB=2 |  |  |
| ACB=3 |  |  |
| **Assessment of cognitive functions** |  |  |
| **s-MoCA score** |  |  |

***CAIDE score*** *(Cardiovascular Risk Factors, Aging and Incidence of Dementia) Dementia Risk Score: 0-9 points is low and normal risk of cognitive impairment development.* ***ACB score*** *(Anticholinergic Burden Score): ≥ 3 points is a higher risk of confusion, falls and death.* ***s-MoCA score*** *(short version of the Montreal Cognitive Assessment): 0-11 normal cognitive functions, ≥ 12 points cognitive impairment.*

**Recommendations given to patient:**

**󠅗 patient´s education 󠅗 management of risk factors 󠅗 retesting in pharmacy – after 6 months**

**󠅗 visit to the physician**

**󠅗 OTC drug/food supplements: (Specified) ...**.......................................................................................................

**󠅗 other:** ...............................................................................................................................................................................................................................................................................................................................................................................................................................................................................................................................................................

**Pharmaceutical counselling in pharmacy date:** ..................................................................

**Pharmacist´s name:**  ..................................................................

**Pharmacist´s signature:** ..................................................................
